# Studying grant decision-making: a linguistic analysis of review reports

**DOI:** 10.1007/s11192-018-2848-x

**Published:** 2018-07-13

**Authors:** Peter van den Besselaar, Ulf Sandström, Hélène Schiffbaenker

**Affiliations:** 1 Network Institute and Dept Organization Sciences, Vrije Universiteit Amsterdam, Amsterdam, The Netherlands; 2 TMC, Amsterdam, The Netherlands; 3 Dept INDEK, KTH Royal Institute of Technology, Stockholm, Sweden; 4 TIP - Technology Innovation Policy Consulting, Joanneum Research, Vienna, Austria

**Keywords:** Peer review, Panel review, Research grants, Decision-making, Linguistics, LIWC, European Research Council (ERC)

## Abstract

Peer and panel review are the dominant forms of grant decision-making, despite its serious weaknesses as shown by many studies. This paper contributes to the understanding of the grant selection process through a linguistic analysis of the review reports. We reconstruct in that way several aspects of the evaluation and selection process: what dimensions of the proposal are discussed during the process and how, and what distinguishes between the successful and non-successful applications? We combine the linguistic findings with interviews with panel members and with bibliometric performance scores of applicants. The former gives the context, and the latter helps to interpret the linguistic findings. The analysis shows that the performance of the applicant and the content of the proposed study are assessed with the same categories, suggesting that the panelists actually do not make a difference between past performance and promising new research ideas. The analysis also suggests that the panels focus on rejecting the applications by searching for weak points, and not on finding the high-risk/high-gain groundbreaking ideas that may be in the proposal. This may easily result in sub-optimal selections, in low predictive validity, and in bias.

## Introduction: the problem with peer review

Peer review in various forms dominates most selection processes within science, although ample evidence shows that peer review suffers from lack of reliability and predictive validity (Chubin and Hackett 1990; Cole and Cole 1981; Bornmann 2011; Van den Besselaar and Sandström 2015). This is not unexpected, as peer review cannot avoid social dynamics (Van Arensbergen et al. 2014; Olbrecht and Bornmann 2010), subjectivity (Lamont 2009) and cronyism through cognitive proximity (Sandström 2009; Wang and Sandström 2015; Sandström and Van den Besselaar 2018) and social proximity (Sandström and Hällsten 2008; Mom and Van den Besselaar 2018). Peer review also suffers from high levels of uncertainty (Van den Besselaar and Leydesdorff 2009; Bornmann et al. 2010; Kaatz et al. 2014). Furthermore, review panel members are asked to apply criteria that are often not or only weakly specified in the funder’s guidelines. Whether reviewers do apply these criteria at all, and how they score and weigh criteria remains invisible and under-investigated.

Reviewing and selecting are social processes, as qualitative studies show (Lamont 2009). Private considerations, heuristics, and (often unconscious) stereotypes do play a role, and these differ from the formal criteria. Heuristics and stereotyping are even more important in decision making under high workload and time pressure (Chugh 2004; Duguid and Thomas-Hunt 2015; Kulik et al. 2000), as is nowadays generally the case in grant allocation procedures. Reviewers are in such circumstances more inclined to use fast heuristics than in depth assessing each application. Despite these problems, peer review keeps on being dominant in grant allocation, and studying peer review remains important as it may contribute to the improvement of the quality of selection processes in science. Our interviews with panel members show that they regularly have strong doubts about the quality of the selection process, so this seems an urgent problem.

## Analyzing evaluative texts

Although it is in principle possible to observe panel deliberation and decision-making, in practice the panel meetings remain closed for investigation.^1^ An alternative strategy is to use *documents* that were produced during the selection process, especially the review reports the applicants receive after the evaluation of their application is finished. The evaluation reports are confidential, but we were able to get permission of about 95% of the applicants to use these for our study. In this paper we present a linguistic analysis of the reviews.^2^

Language embodies normative views about who/where we communicate about, and stereotypes about others are embedded and reproduced in language (Burgers and Beukeboom 2016; Beukeboom and Burgers 2017). Linguistic analysis techniques have become increasingly powerful for analyzing evaluative texts, such as reports of annual performance interviews (Semin and Fiedler 1991; Biernat et al. 2012; Kaatz et al. 2015), and tools for supporting such analysis are becoming available (Ignatow and Mihalcea 2017). A main focus in the study of evaluative texts is the presence of bias in decision-making, such as gender bias (Biernat et al. 2012; Kaatz et al. 2015), and quite some empirical support is available for the gender-stereotyping practices in hiring and promotion in general, but also in hiring, promoting, and grant decisions within science (Miller et al. 2015). Sentiment analysis may be used to identify the opinions and feelings of the authors. More specifically, one may detect whether positive or negative emotions are be connected to certain aspects of the proposal, e.g., the track record of the applicant, the risk level of the proposal, or more generally to the reputation of the applicant or of the environment (e.g., a top or average university) the applicant is affiliated with. In this paper, we use linguistic analysis of the review reports for assessing the quality of peer and panel review for grant decision-making. More specifically, this paper answers the following questions: (1) What is discussed about proposals within the panels? (2) What can we learn about the way panels work? (3) What are the strengths and weaknesses of a linguistic approach? More specifically, we will try to find out what criteria are deployed and how they are deployed.

## The case

The ERC Starting Grants are probably the most prestigious grant for early career researchers. The grant is large (1.5–2 million euros for a 5-year period) and open for researchers worldwide. The constraint is only that the research will be done at an EU university or research organization. Competition is high, and success rate is low (slightly above 10%).

In the 2014 round—our case—there were about 3200 applications, which are assessed in 25 disciplinary panels—ten for Physics and Engineering, nine for Life Sciences, and six for Social Sciences and Humanities. We will not go into field differences here, but field differences may also be reflected in language use, and in the embedded stereotypes (Leslie et al. 2015). A typical panel consists of about ten panel members, and handles between 80 and 250 applicants. The 25 panels discuss and evaluate the 3200 applicants in about 2 days, implying less than 10 min per proposal. According to several interviewees, excellent and low-quality applications are generally decided without much discussion, so there remains more (but not much) time for the less clear cases. So, time pressure remains an important problem. From research on group decision-making, it is well known that group members under (time) pressure start to use heuristics and stereotypes, and do not necessarily assess every case in a systematic way (Van Arensbergen et al. 2014). Consequently, bias may come in easily. If this is the case, one would expect that the panel scores and the panelists’ word use in the review may only correlate weakly with more objective quality indicators.

In the first phase of the selection process, panels assess five-page proposals plus a five-page CV, generally without making use of external peers who are specialists with respect to the proposed research. In the second round, additional-up to nine, and often external-reviewers are included in the assessment process of an extended version of the proposal.

The panel members are instructed by the president of the council on how to do the selection process. ‘Excellence’ should be the only criterion, but what this means remains rather unspecific (Schiffbaenker and Haas 2018). Some characterizations of ‘excellence’ are suggested, but they are not operationalized (ERC European Research Council 2013):

- Projects should be potentially groundbreaking, high risk–high gain, and preferably interdisciplinary.
- Applicants should have developed independence.
- Applicants should be able to do to groundbreaking research and have shown that in the past.

Only for independence some yardstick is formulated: Applicants should have publications not coauthored with the Ph.D. supervisor. The low codification of the criteria is intentional, as the ERC wants to give panel members the freedom to do the selection in their own way. The selected panel members are seen as the leading scholars in their fields, and therefore know the best how and what to select.

## Methodology

### Data

We interviewed 32 panel members. We focused on four panels in order to investigate at the panel level the degree of agreement about what criteria should be used, and how they should be operationalized. We interviewed the panelist also about the review process in order to identify possible problems in and the quality of the selection process. We used open interviews and use some of the results in this paper. A systematic analysis of the interviews will be done in another paper.

We got access to the project descriptions, CVs, reviewer scores, and review reports of 3030 applicants. We retrieved bibliometric data for (at the moment of writing of this paper) some 1200 applicants. For the linguistic analysis, we use the review reports of 3030 applications, of which 352 were successful and 2674 rejected. These 3030 cover more than 95%^3^ of all applications in the 2014 Starting Grant call. In the first selection phase, 75% of the applicants are rejected. In the second phase the winners are selected,^4^ which are 45% of those that made it to phase 2.

The review reports form the core input for the panel discussion, and for the summary report produced after the deliberations. In the first round of the selection process, the 75% of the proposals have four reviews, in 10% of the cases three, and in 12% five reviews. A few had six or seven reviews. Those that make it to the second round are generally evaluated by six, seven or eight reviewers (almost 80%), but again there are cases with more (in a few cases even thirteen) reviewers. The analysis presented here combines the (rather short) review reports about a proposal into a single document. Automatic pre-processing was done to remove the irrelevant text: instruction sentences, section headings, etc. For some samples, we checked manually whether the conversion from PDF into TXT worked correctly.

Each reviewer gives a score for the PI and a score for the project in both phases of the selection process. For the analysis in this paper, we average the reviewers’ scores, so in both phases, the applications have one PI-score and one Project-score. We calculate for each proposal the coefficient of variation for the scores, as indicator for the level of consensus about the proposal.

### Analytical instruments

LIWC (http://liwc.wpengine.com/) is a tool for linguistic analysis of texts, based on a variety of predefined linguistic categories. Each category consists of a series of words, and the linguistic categories are validated in other studies (e.g. Abele and Wojciszke 2014). The LIWC program counts for each of the categories how many times a word belonging to that category is present in a document. As documents are of different length, normalization is needed: the frequency is translated into a percentage.

For our study, we firstly selected the same LIWC-categories used by Kaatz et al. (2015). As that is a (small scale) US-based study, we assume that word use may be partly different. More specifically, the specific evaluative words used in the grant scheme under study need to be added. We retrieved the 10,000 most frequently occurring terms in the review reports, to identify case specific words that had to be added to the categories. The selection was done in parallel by a researcher and a research assistant. Where the selections were different, we discussed what terms should or shouldn’t be included.

Compared to Kaatz et al. (2015), we split one category into two, and added a few more categories from the LIWC dictionary. The *research* category refers to the track record of the PI and to the quality of the research proposal, which are two different evaluation categories in the case under study. We therefore separated *research* into *track record* and *proposal.*

Based on insights in communication theory (Burgers and Beukeboom 2016) we added the following categories. Firstly *negation* words, as excellent applicants are the norm in science, and the ‘others’ are measured against those excellent: ‘not excellent’. One would therefore expect much more negation words in evaluation reports of non-successful applicants than in those of successful applicants. *Exclusion* words might be used differently because of the same argument: more used for proposals and applicants that meet hesitation. *Certainty words* are also informative about the process: one would expect that the very good and the rather low-quality proposals meet little uncertainty, whereas this is different for the average applicants. *Positive and negative emotions* are relevant to include, as one would want to see how strong sentiments play a role in panel deliberation. This leads to the next list of linguistic categories:^5^

1. *Negative evaluation* words such as *naïve*, defect*,^6^ lack* (Kaatz et al. 2015).
2. *Positive evaluation* words like *intriguing*, *compelling*, commit* (Kaatz et al. 2015).
3. *Superlatives*^7^ *such as world class*, *outstanding*, exceptional*, groundbreaking, *grand challenge*, *forefront*, *great potential*, *high risk high gain* (LIWC 3% extended).
4. *Track record* words such as result*, fund*, high impact journal, *coauthor*, *H*-*index*, *editor*, *advisor* (part of LIWC-research, 9% extended).
5. *Proposal* words such as *laboratory, multidisciplinary*, *timeline* (part of LIWC-research, 9% extended).
6. *Ability* words, such as gift*, intell*, skill* (LIWC 10% extended).
7. *Achievement* words^8^ such as creati*, excel*, compet* (LIWC 20% extended).
8. *Agentic* words such as *outspoken*, *solid*, *risk* (LIWC).
9. *Negating* words such as *hasn’t*, *don’t*, *can’t* (LIWC).
10. *Exclusion* words such as *but*, *either*, *except*, *just*, *not* (LIWC).
11. *Certainty* words such as *fundamental*, *commitment*, *truly* (LIWC).
12. *Negative emotions* words such as abuse*, bitter*, bad* (LIWC).
13. *Positive* emotions words such as agreeabl*, *benefit*, *helpful* (LIWC).

### Method

In order to contextualize the linguistic analysis of the review reports, we inspected for a sample the structure and the content of the review reports. This was done to detect the level of systematic assessment using the ERC dimensions and criteria.

The linguistic analysis (LIWC) gives for every review the percentage of words belonging to each linguistic category. With these, one may compare the average frequencies of the categories between those that won in the first phase and those that did not, and between those that were accepted for funding in the second phase and those that were not. The results will inform us which word categories are related to success and which to the opposite.

Then we deploy linear regression to predict the panel scores from the linguistic categories. From this, we may learn which quality dimensions are relevant and in which order of importance.

## The decision-making process

As indicated above, there are a few criteria mentioned in ERC policy reports (ERC 2013), such as independence and the ability to do groundbreaking research, but nowhere in the documents these criteria are specified or translated into operational criteria, giving individual reviewers all leeway to define the criteria in their own way. This gives panels and panel members freedom to choose their own approach for *selecting excellence only*, as the mentioned criteria for evaluating the PI are unspecific: what is ‘independent’ and how can a reviewer see this? And what is ‘ability to do groundbreaking research’? Is that having published in Nature, having published a very highly cited paper, or something else?

This is confirmed by reading through a sample of review reports. There one regularly finds arguments that go in fact another direction than the ERC guidelines suggest. Quite a few examples were found where risk of a project was seen as negative, even when the gains in case of success would be high. For example, one of the panels writes “if the project would be successful, it would really change the field”. The panel seems to hesitate whether the field would take up the results of the project, which they call “a risk of the project”. This is clearly a high risk–high gain proposal, but the conclusion is that “the project may probably fail” and it gets low scores—even not high enough to go to the second phase.

Also the interviews suggest that panelists assess in different ways (Schiffbaenker and Haas 2018), and they are also critical about the approaches of other panel members. Such as a panelist who tells that he looks at the quality of individual papers, and at highly cited papers. Several of his colleague panelists, he explains, do it differently: they look at journal impact factors, that according to the interviewed panelist “do not tell anything about the individual papers of the applicant”. If it is unclear how to assess the PI, this holds even stronger for the assessment of the proposed research project. For the project the only relevant criterion is ‘excellence’, but no effort was made by the ERC to specify this is any operational way at all.

This lack of specific and operational criteria is a general problem in the evaluation of proposals, as we found when interviewing some 30 panel members. Most panel members pointed out that due to a lack of specification, applying criteria and choosing indicators is a challenge that demands individual strategies:

They give you very general guidelines like the scientific quality, the quality of the researcher, the originality of the proposal, and so on, typical of all projects. In those projects that are so related to your field of expertise you don’t even need it because you appreciate them immediately. The problem comes when the projects are far from your field of expertise, then you have to be very objective in your criteria, so I have prepared a list of things I should not be forgetting. (Panel member 17, LS, female)

That this is a real problem, relates to the way the review process is organized. The panels are relatively broad, and the about 10 members per panel have to cover several fields and many research fronts. For example, panel PE1 covers mathematics and computer science, and as quite some mathematicians also publish on mathematical physics, PE1 panelists also have to cover the latter field. Panel members evaluate proposals that are not within their direct field of expertise, and according to some interviewed panelists, even topics and fields rather far away from their own field. Therefore, what is called *peer review* is in many cases in fact *expert review*: Reviewers are experts, but not necessary in the research topic of the applicant.

The lack of well-defined indicators was particularly articulated for assessing the PI: “For the PI the criteria do not work at all. They should be much clearer, much more objective.” (Panel member 32, SH, female). This challenge was also found in earlier studies. In the Norwegian context “panel members had no problem in pointing out criteria for judging scientific quality, but they had problems explaining how they *use* [italics in origin] these criteria” (Langfeldt 2004, 57).

In the current case, only a few clear indicators are defined, but these are not perceived as binding for the assessment process. Consequently, very different achievements of applicants are taken into account when a specific criterion is operationalized. For example, the indicator for the applicant’s independence is ‘having at least one important publication without the Ph.D. supervisor’. But some of the interviewed panel members don’t consider this indicator at all, and mention other indicators for independence like having acquired funding, the composition of the team the applicant is leading, the topical distance to the former Ph.D. supervisor, having worked in different institutions, or having established new research collaborations. Apart from the question whether these are a valid operationalization of ‘independence’, the main finding is that different panel members mention and probably use rather different indicators for assessing the same element of excellence.

Finally, the interviews indicate that different panel members’ focus on different dimensions of excellence. For some panel members, it is the research idea that is most important, for others it is the track record, and for again others it is the quality of the journals the applicant has published in. The use of different excellence dimensions results in *intransitivity*: it makes the comparison of applicants in a meaningful way impossible. Therefore, it may result in inconsistencies within and across different selection rounds, and into suboptimal outcomes (Herschberg et al. 2014). Obviously, the lack of guidelines for operationalizing excellence leads to a unsystematic use of criteria and indicators, which may easily result in biased evaluation practices.

## The linguistic analysis

The interviews suggest a rather unsystematic evaluation process, but what can we learn about it from the review documents about the process and the criteria deployed? In order to answer that question, we focus on the linguistic categories that are used in the review reports. The analysis shows that words referring to the *proposal* and to the *achievements of the PI* are most frequently used. Also *positive evaluation* words, *positive emotion* words, and *superlatives* appear rather frequent. Lower in the list are most of the negative terms: *Exclusion* words, *negative evaluation* words, *negation* words and *negative emotion* words. Among the less-frequently used categories we also find *agentic* words, and *track record* related words (Table 1).

**Table 1:** Mean frequency of linguistic category

Word count	1394	Agentic	1.81
Proposal^a^	6.74	Negative evaluation	1.76
Positive evaluation	4.31	Track record^a^	1.42
Achievement	3.94	Negation words	1.36
Positive emotions	2.91	Negative emotions	1.00
Superlatives^a^	2.00	Ability	0.58
Exclusion	1.92	Certainty	0.25

Average % words in the review reports belonging to a linguistic category

^a^Including the ‘typical ERC words’ in the review reports

We now firstly compare the successful applications with the non-successful ones, in both phases of the selection process, in terms of the word categories used in the evaluation reports. Are some linguistic categories much more or much less frequently used for the successful applications? Secondly, we use the linguistic variables to predict the scores received from the panels. Together this may inform which evaluation dimensions are more important and discriminate between success and rejection, and which are less important.

### Success versus no success in phase 1 and in phase 2

Table 2 shows the *ratio* of *average occurrence* of the linguistic categories: (1) the accepted versus the rejected applications in phase 1; (2) the granted versus non-granted applicants in phase 2. Review reports of applicants that are successful in phase 1 show significantly more certainty words, agentic words, ability words, superlatives, achievement words, positive evaluation words and positive emotion words, and *significantly less* negation words, exclusion words, negative evaluation words (Table 2, left side). Intuitively this seems a reasonable result. When the track record and the research proposal are addressed more extensively in the panel report, it is obviously more often with a negative effect. This is in line with the theory of Festinger, suggesting that more words imply more disagreement: researchers are inclined to talk more about things they disagree about (Festinger 1950; Buttliere 2015, 2017). So, when the panel members talk more about the research project and the track record they disagree more on the quality, and this leads to lower scores.

**Table 2:** Relative frequency of word category use

PHASE 1 Accept/reject*	Ratio***	Sig.	PHASE 2 Granted/rejected**	Ratio***	Sig.
Certainty	1.38	0			
Agentic	1.08	0	Agentic	1.04	0.019
Ability	1.07	0.011			
Achievement	1.05	0	Achievement	1.04	0.008
Superlatives	1.04	0.002	Superlatives	1.08	0
Positive emotions	1.03	0.017	Positive evaluation	1.04	0.006
Positive evaluation	1.03	0.004	Positive emotions	1.05	0.003
			Negative emotions	0.93	0.020
Proposal	0.92	0			
Track record	0.75	0			
Exclusion	0.69	0	Exclusion	0.88	0
Negative evaluation	0.65	0	Negative evaluation	0.83	0
Negation	0.57	0	Negation	0.79	0

ERC starting grants 2014, 3030 applicants

*N = 785 resp. 2241; **N = 352 resp. 433

***Ratio = ratio of the mean word frequency of the two groups, and the significance level comes from an Anova test. Ratios > 1 mean that the linguistic category occurs more in review reports about those that are successful; ratios < 1 mean that the linguistic category occurs more often in review reports about applicants that were rejected. Non-parametric tests show a similar result

Comparing granted versus the rejected applications in the second phase (Table 2, right side), less linguistic categories show a statistically significant different frequency between the two groups. This is at least partly due to the much lower N in phase 2. Especially most of the categories pointing at the performance (track record), the characteristics of the applicant (ability) and the application (proposal) do not appear anymore as different, and negative emptions enter the list. But the more general linguistic categories remain significant, similarly to the pattern found in phase 1: In both phases, the negation and negative evaluation words show the strongest difference. As success rate is higher in the second phase, one would expect differences being smaller—which is indeed the case.

What does this suggest about the deployed decision dimensions? Most of the linguistic categories refer to the assessment terms (superlatives, positive evaluation, negative evaluation, negation words) and the feelings of panel members (emotion words). Some others refer to *past performance of the applicants* (achievement words, track record words), to *personal characteristics* (agentic words, ability words), and to *quality of the proposed project* (proposal words). The latter three reflect the *dimensions* (more than operational criteria) that play a role in the decision-making: The ERC asks for a score on the project, and on the PI, and the latter consists of past performance, and the ability to do excellent research. As Table 2 suggests, more writing about the person has a positive effect, more writing about the project and the past performance has a negative effect.

### Evaluating the project and evaluating the applicant

As mentioned above, the panels should evaluate the quality of the project proposal and the quality of the applicant separately. In theory, these are two different quality dimensions. However, the panel scores for the project and for the PI correlate very strong. In the first phase of the procedure, the correlation between the score for the PI (based on the CV) and the score for the project (the ideas in the project description) is 0.87, which shows that the panel members do not distinguish between those two dimensions, although the ERC claims that both are critical in the evaluation procedure. We would propose the hypothesis that panel members in fact focus on one dimension and then adapt the score for the other dimension.^9^ This being the case, one would expect that the same linguistic categories predict the two scores. This is investigated in the next section.

### What evaluation dimensions predict the scores?

In the next step, we predict the two scores in the first phase (PI-1 score and Project-1 score) and the scores in the second phase (PI-2 score and Project-2 score) by the frequencies of the linguistic categories as the independent variables. As this is an explorative study, we deploy linear regression with a *backward removal* of the independent variables.^10^ This leads to a model in which the non-significant variables are excluded. As the linguistic categories may be used not independently, we tested for multicollinearity. Tolerance and VIF values suggest that this does not occur. We assume that disagreement in the panel has an effect too—disagreement may avoid high scores. We control for disagreement through the *coefficient of variation* of the scores of the different panelists, and the average CoV for successful applicants is about 15% lower than for the non-successful.

We start with the prediction of the PI-score in phase 1. Table 3 shows the linguistic categories that remain in the (final) model. We report here also the standardized regression coefficients, as that enables to assess which of the variables has the strongest effect. The negation variable and the *negative evaluation* variable have the strongest effects on the score.^11^ The *superlatives* is the next strongest variable. All the other categories have small(er) effects: words referring to the research *proposal*, to the *track record*, and to *achievements* have a negative correlation with the score, suggesting that when the panel talks about the proposed project and the track record, this is more often in a negative than in a positive way.

**Table 3:** PI-score (first phase) by frequency of linguistic categories

Model 5	Unstandardized		Standardized		Sig.	95.0% Conf interv for *B*	
	*B*	SE	Beta	*t*		Lower Bnd	Upper Bnd
(Constant)	3.423	0.062		55.151	0	3.301	3.545
Negation	− 0.453	0.028	− 0.472	− 16.18	0	− 0.508	− 0.398
Negative evaluation	− 0.162	0.021	− 0.189	− 7.561	0	− 0.204	− 0.12
Superlatives	0.089	0.015	0.100	5.818	0	0.059	0.119
Proposal	− 0.039	0.006	− 0.092	− 6.441	0	− 0.051	− 0.027
Track record	− 0.062	0.010	− 0.088	− 6.072	0	− 0.082	− 0.042
Certainty	0.258	0.044	0.081	5.841	0	0.171	0.345
Disagreement	− 0.19	0.039	− 0.066	− 4.923	0	− 0.266	− 0.115
Exclusion	0.053	0.022	0.064	2.426	0.015	0.01	0.096
Positive evaluation	0.035	0.011	0.056	3.179	0.001	0.013	0.057
Positive emotions	0.038	0.012	0.050	3.27	0.001	0.015	0.061
Adjusted *R*^2^ = 0.463							

The other categories have significant positive relations with the score, but the effect of all is rather small, and especially much smaller than the effect of the negation and negative evaluation category. Interesting is that negative emotions has no effect, and the positive emotions only a very small one. Finally, exclusion, negative emotions, agentic and ability words have no significant effect. The model has an *R*^2^ of 0.463, which means that the variance is almost half explained by the linguistic variables. Disagreement between the panelists (measured through the variation in the panelists’ scores) works negatively too. The results for the project score (Table 4) and the PI score are rather similar. Also for the project score, the explained variance is quite high (0.507), even slightly higher than for the PI score. *Achievement*, *agentic* and *ability* words are removed from the analysis as they did not have a significant effect. In this analysis *negative emotions* come in but with a rather small (negative) effect on the project score.

**Table 4:** Project-score (first phase) by frequency of linguistic categories

Model 5	Unstandardized		Standardized		Sig.	95.0% Conf interv for *B*	
	*B*	SE	Beta	*t*		Lower Bnd	Upper Bnd
(Constant)	3.337	0.058		57.91	0	3.224	3.450
Negation	− 0.487	0.025	− 0.544	− 19.446	0	− 0.536	− 0.438
Negative evaluation	− 0.159	0.019	− 0.197	− 8.24	0	− 0.196	− 0.121
Exclusion	0.093	0.02	0.121	4.756	0	0.055	0.131
Superlatives	0.095	0.014	0.115	6.942	0	0.068	0.121
Proposal	− 0.041	0.005	− 0.102	− 7.429	0	− 0.051	− 0.03
Track record	− 0.056	0.009	− 0.085	− 6.065	0	− 0.074	− 0.038
Certainty	0.193	0.04	0.065	4.884	0	0.116	0.271
Disagreement	− 0.147	0.032	− 0.059	− 4.588	0	− 0.209	− 0.084
Positive evaluation	0.033	0.01	0.056	3.334	0.001	0.014	0.052
Positive emotions	0.035	0.01	0.050	3.396	0.001	0.015	0.056
Negative emotions	− 0.031	0.014	− 0.029	− 2.203	0.028	− 0.058	− 0.003
Adjusted *R*^2^ = 0.507							

We now do the same for the second phase, where the success rate is much higher than in the first phase: about 45% of the applications that make it to the second phase do get the grant. One may expect that all applications and applicants in phase 2 are of exceptional quality—which means that the argumentation in this phase of the selection process would be different from the discourse in the first phase. Consequently, one would expect other linguistic dimensions becoming dominant. This is indeed the case. As Table 5 shows, in phase 2 *negation words* remain influential, but now *panel disagreement* has a very strong negative effect. On the positive side, we find *superlatives*, and with a much smaller beta, *positive emotion* words. The project-score (Table 6) is first of all influenced by linguistic categories with a negative effect: *disagreement, negation words, track record words* and *negative evaluations*, and here *superlatives, positive emotions* and *ability* have small positive effects.

**Table 5:** PI-score (second phase) by frequency of linguistic categories

Model 4	Unstandardized		Standardized		Sig.	95.0% Conf interv for *B*	
	*B*	SE	Beta	*t*		Lower Bnd	Upper Bnd
(Constant)	3.778	0.074		50.882	0	3.632	3.924
Disagreement	− 0.755	0.051	− 0.433	− 14.889	0	− 0.854	− 0.655
Superlatives	0.113	0.021	0.218	5.340	0	0.072	0.155
Negation	− 0.172	0.046	− 0.174	− 3.717	0	− 0.263	− 0.081
Track record	− 0.065	0.012	− 0.176	− 5.609	0	− 0.087	− 0.042
Exclusion	− 0.093	0.034	− 0.125	− 2.726	0.007	− 0.160	− 0.026
Positive evaluation	− 0.032	0.015	− 0.085	− 2.132	0.033	− 0.062	− 0.003
Positive emotions	0.032	0.015	0.073	2.219	0.027	0.004	0.061
Adjusted *R*^2^ = 0.387

**Table 6:** Project-score (second phase) by frequency of linguistic categories

Model 4	Unstandardized		Standardized		Sig.	95.0% Conf interv for *B*	
	*B*	SE	Beta	*t*		Lower Bnd	Upper Bnd
(Constant)	3.576	0.066		54.117	0	3.446	3.705
Disagreement	− 0.51	0.046	− 0.304	− 11.082	0	− 0.601	− 0.42
Negation	− 0.314	0.04	− 0.300	− 7.851	0	− 0.392	− 0.235
Track record	− 0.094	0.011	− 0.243	− 8.225	0	− 0.116	− 0.072
Negative evaluations	− 0.136	0.031	− 0.166	− 4.361	0	− 0.198	− 0.075
Superlatives	0.07	0.017	0.127	4.115	0	0.037	0.103
Positive emotions	0.055	0.014	0.117	3.814	0	0.027	0.083
Ability	0.074	0.029	0.067	2.52	0.012	0.016	0.132
Adjusted *R*^2^ = 0.440							

Summarizing the findings, we found differences and similarities between the two phases in the decision-making process. The *negation words* have in about all cases the strongest effect, so the more negation words, the lower the score by the panel. And in the first phase, this is reinforced by *negative evaluation* words. In both phases, the more *superlatives* the higher the panel score, but the related beta is (much) smaller than the beta of the negation words. Everywhere, *panel disagreement* has a negative effect on the score, but in the second phase this effect is strong, especially for the PI-score. Finally, in the first phase, the more words referring to the *proposal* and to the *track record* of the PI, the lower the score. Overall, in both phases, the variables that predict the PI-score are similar to those that predict the Project-score. As panel disagreement has such a strong effect in the decisive second phase, one wonders whether language is  reflecting disagreement. We tested this, and the positive evaluation and superlatives correlate negatively with panel disagreement and the negation words and negative evaluation words correlate positively but weakly with panel disagreement.

## How are evaluation reports related to performance indicators?

To understand the meaning of the linguistic variables somewhat better, we investigate the relation of these variables to performance indicators. After cleaning the bibliometric data for four life sciences panels, scores for a set of indicators were calculated. We use here the bibliometric indicators that have—within the life sciences domain—a significant effect on the final score the applicants got (Van den Besselaar et al. 2018): the normalized journal citation score (NJCS) reflecting the impact factors of the journals published in; the share top 5% most cited papers; the number of grants received, and the quality of the collaboration network. The latter is based on the median pp10% score of the organizations mentioned in the CV. We also include the number of international coauthors and the number of all coauthors. Table 7 shows the correlation between the performance scores and the linguistic variables.

- The ‘positive’ linguistic categories (positive evaluation; superlatives; certainty; positive emotions) correlate weak but positively with the performance variables.
- The ‘negative’ categories (negative evaluation; negation; exclusion) correlate moderately negative with the performance variables. As the ‘negative’ linguistic categories have a stronger effect on the panel scores, the panel scores seem to reflect the opinion about the past performance.
- The two linguistic categories referring to the proposal and to the track record of the applicant show the same pattern as the negative linguistic categories. If the applicant has lower bibliometric scores, there is more attention in the review for track record and proposal. As we showed above, this results in a lower score for project and PI in the first decision making phase.
- The categories referring to personal characteristics (achievement; agentic) correlate negatively with the number of co-authors. The same holds for the positive emotions, track record and superlatives. Despite the increased role of team science, a high number of coauthors does not work positively. For international coauthors, the patters seems opposite: that seems assessed positively.

**Table 7:** Linguistic categories by performance scores

	NJCS journal impact	Top 5% cited papers	# Grants	Quality network	# Intern. co-authors	# Co-authors
Positive evaluation		0.151^**^		0.136^*^		
Superlatives			0.134^**^	0.162^**^		− 0.114^*^
Certainty	0.113^*^	0.111^*^				0.116^*^
Positive emotion					0.108^*^	− 0.214^**^
Negative evaluation	− 0.304^**^	− 0.199^**^	− 0.186^**^	− 0.267^**^	− 0.108^*^	
Negation	− 0.334^**^	− 0.234^**^	− 0.197^**^	− 0.302^**^	− 0.121^*^	
Exclusion	− 0.257^**^	− 0.170^**^	− 0.134^*^	− 0.239^**^		
Track record	− 0.197^**^	− 0.135^**^		− 0.175^**^		− 0.341^**^
Proposal	− 0.171^**^	− 0.103^*^		− 0.127^*^		0.119^*^
Agentic				0.131^*^		− 0.193^**^
Achievement						− 0.187^**^

Four life science panels, phase 1: *N *= 348;

*Significant at the 0.05 level (2-tailed)

**Significant at the 0.01 level (2-tailed)

Overall, the correlation between the linguistic variables and the variables measuring past performance and the collaboration network are weak to moderate. This supports the finding that panel members have individual ways of assessing the quality of an application. On the other hand, the direction of the correlations are in line with the findings in the linguistic analysis.

## Conclusions and discussion

Interview results and reading review reports indicates that peer and panel review is weakly codified. Panel members emphasized the lack of clear and operational criteria to assess applicants and project proposals. From the interviews it also became clear that different criteria are used by different reviewers, and this may negatively influence the quality of the selection process. The review reports support this, as they lack a format in which different criteria are systematically scored. Also, the (few) explicit evaluation criteria and dimensions (high risk high gain; groundbreaking; etc.) are not systematically developed nor deployed.

The linguistic analysis of the review reports brought some interesting findings, showing how studying review reports can inform us about the processes and problems in peer review processes, and to some extent it identifies the relevance of specific evaluation dimensions in grant-decision making. In contrast to other studies, we do have a large sample, and this may lead to more reliable and valid results.^12^ And we included next to the subjective panel scores some objective performance criteria that can function as frame of reference when evaluating the panel assessment (Van den Besselaar and Sandström 2015, 2016).

What does the linguistic analysis show? The strongest effect on the scores—for the PI and for the project—comes from the negative linguistic categories: negation words, negative evaluations, and exclusion words. This suggests that panels concentrate in the discussions on identifying the (in their opinion) weakest proposals (and rejecting those) instead of trying to find the best and promising ones. Where panels discuss more intensively the track record of the PI and the quality of the proposal, it is more often in a negative than in a positive way—as we can conclude from the negative relation between the prevalence of those linguistic categories and the scores. Concluding, the main strategy is reducing the pool of applications.

What lies behind this negative strategy of getting rid of applications? There are two factors that may cause this to happen (related to group decision making theory): (1) the enormous selection pressure as there is a very low success rate of about 10%, and (2) the enormous work load, as a panel has to process on average 100 proposals in 2–3 days. There is on average less than 10 min for discussing an application—proposed project and the applicants’ CV. As we know from literature, and also found in interviews, this results in heuristic decision making. On top of this comes that, according to an interviewee, panel members also have to review and discuss research proposals that are very far from their expertise. And as showed elsewhere (Bornmann et al. 2010), under such conditions there is quite a considerable chance of ‘false negatives’ and of ‘false positives’: many excellent applicants and applications may be rejected too soon, and some not so good but well-presented may be accepted too soon. To some extent, reducing by eliminating may be useful, as there are always quite some not so good proposals, but when this should lead to a rejection of 75% of the applications, one asks too much of a selection process with a high level of inherent uncertainty.

We also found that the same linguistic factors play a role when scoring the PI as when scoring the proposal. That should in fact not be the case as some linguistic categories refer to the person, and others to the project. This is in line with another finding: the panel scores for the PI and for the project correlate very highly—it seems that panel members do not or cannot distinguish between the two evaluation dimensions. This is also in line with the correlation between the linguistic variables with some (objective) performance scores. The negative linguistic categories are dominant in explaining the scores, and moderately (negative) correlated with the performance variables.

The study has a few limitations that should be mentioned here and that at the same time point at further research. First of all, the reviews are edited in the sense that rough language is deleted, and that inconsistency between text and scores are corrected. For several panels, we also inspected the un-edited comments, and this gives the impression that the editing does not affect the results of this study. However, this is something that requires further research. Secondly, within the context of this study we cannot test the quality of the original and the modified dictionary. Changing the dictionary will of course influence the results, and should do so. Our additions, however, were needed to link the LIWC dictionary to the specific language used in the context of the council under study. Finally, as grading and decision-making takes place within panels, we may add this as an additional level in a multi-level analysis of the data. It also would be highly relevant to add other variables to the analysis, such as gender. That would help investigating whether male and female applicants are evaluated in different language, and whether these language differences reflect for example gender stereotyping (Miller et al. 2015; Kaatz et al. 2015)?

## Footnotes

1 In the research proposal for this project, we included panel observations. However, observations were not possible in the end, as too many panel members did not agree. Only a few observation studies are available. A Swedish study shows considerable problems concerning gender equality in The Swedish Research Council’s evaluation panels (see Vetenskapsrådet 2013). At CNRS (France), researchers were allowed to observe the interview of the applicant buy the panel, but not the deliberations before and after the interview (Presentation Anne Pepin at GEHE 2016 in Paris). Our own (small scale) experiences, however, suggest that these deliberations are crucial in decision making.

2 Review reports are edited—rough language is taken out not to insult the applicants, and inconsistencies and unclear statements that may lead to appeal are corrected.

3 About 5% of applicants did not give permission to use their data for research.

4 Formally it is not the panel that decides, but the ERC scientific council.

5 For more details about the dictionary please contact the corresponding author. For more information about the LIWC software and dictionary: pennebaker@utexas.edu.

6 The * after words indicate that words appear in different forms.

7 This is an extension of the LIWC *Standout adjectives* category with a list of ERC specific superlatives.

8 To what extent should we split the categories into positive and negative? E.g., masculine is an *agentic* word, but energetic is an *agentic* word too. We have done such a test for positive versus negative *achievement words* and that makes no difference to only achievement words.

9 It would be easy to solve this, e.g., by having some panel members evaluating the project (without having the CV) and other panel members evaluating the PI (without having the project description).

10 Criteria: P-in = 0.49, P-out = 0.50.

11 In fact, the two (and ‘exclusion’) correlate about 0.9 so could be combined into one variable (this critical level of multicollinearity also explains why ‘exclusion has a positive sign).

12 It has been argued that small samples are the main cause of bias in the scholarly literature (Fanelli et al. 2017).
